# Inactivating conditions of therapeutic mycobacteriophages

**DOI:** 10.1101/2025.09.30.679648

**Published:** 2025-09-30

**Authors:** Andrew Wiggins, Umar N. Chaudhry, Fabiana Bisaro, Addison Lueck, Alan A. Schmalstig, Graham F. Hatfull, David B. Hill, Miriam Braunstein

**Affiliations:** 1 Mycobacteria Research Laboratories, Department of Microbiology, Immunology, and Pathology, Colorado State University, Fort Collins, Colorado, USA; 2 Department of Microbiology and Immunology, University of North Carolina at Chapel Hill, Chapel Hill, North Carolina, USA; 3 Cell and Molecular Biology Program, Colorado State University, Fort Collins, Colorado, USA; 4 Department of Biological Sciences, University of Pittsburgh, Pittsburgh, PA, USA; 5 Marsico Lung Institute and Lampe Joint Department of Biomedical Engineering, University of North Carolina at Chapel Hill, Chapel Hill, North Carolina, USA

**Keywords:** Bacteriophage, Nontuberculous Mycobacteria, mycobacteriophage

## Abstract

There is a need for new therapies to treat drug resistant nontuberculous mycobacteria (NTM) disease. Bacteriophages (phages), which are viruses that infect and kill bacteria, are actively being explored as an alternative approach for treating mycobacterial diseases. Several compassionate-use cases of phage therapy for drug resistant NTM infections exhibit favorable outcomes. To further the development of phage therapy it is important to recognize and avoid conditions that negatively impact phage activity during phage production, storage, formulation, or treatment. Conversely, there is a need to inactivate free phages in certain preclinical phage therapy experiments. In this study, we investigated three mycobacteriophages BPsΔ*33*HTH-HRM10, Muddy, and ZoeJΔ*45* from compassionate-use NTM treatment cases for their sensitivity to a variety of conditions that included temperature, acid pH, detergents, mucus, and phage inactivating buffers. Several conditions resulted in dramatic and rapid reductions in the level of active phage while others had no effect. We also observed different sensitivities between the phages. The results provide valuable information to support further investigation and development of these phages as therapeutics.

## Importance

Bacteriophages (phages) offer a promising alternative therapy for treating drug resistant mycobacterial infections. For successful implementation of phage therapy, it is important to recognize conditions that inactivate the phages. Here, we studied three mycobacteriophages from recent compassionate-use phage therapy cases for their sensitivity to a range of conditions that may be encountered in production, storage, formulation, or treatment. The results demonstrate sensitivity to some conditions and tolerance to others, and they additionally reveal phage-specific differences in sensitivities highlighting the need for direct evaluation of individual therapeutic phages during development.

## Observation

As bacteria get harder to treat with antibiotics, the need for alternative therapies becomes more urgent. An alternative to antibiotics is the use of lytic bacteriophages (phages), which infect and kill bacteria. There are a growing number of compassionate-use cases of phages being used therapeutically to treat patients with multidrug-resistant bacterial infections, including nontuberculous mycobacteria (NTM) pulmonary infections (1, 2) For phage therapy to succeed, it is important to avoid conditions that negatively impact phage activity when producing, storing, or formulating phages since low phage titers could jeopardize treatment, as likely occurred in a clinical trial of phages for *Pseudomonas aeruginosa* infections (3–5). Conversely, a method for inactivating phages is useful in preclinical experiments where the bactericidal activity of phages is measured by bacterial CFU (i.e., to avoid free phages from reducing CFU during outgrowth). Here, we evaluated three siphovirus mycobacteriophages from phage therapy cases of NTM infections, each mapping to a different genomic cluster, for their sensitivity to a variety of conditions: BPsΔ*33*HTH-HRM10 (BPsΔ) (cluster G), Muddy (cluster AB), and ZoeJΔ*45* (ZoeJΔ) (cluster K) (6).

To measure phage sensitivities, phages at titers of 10^9^ – 10^10^ PFU/ml in mycobacteriophage (MP) buffer were exposed to different conditions at room temperature (22°C), unless otherwise indicated. At specific time points, phages were quantified as PFU/ml using an agar overlay assay with *Mycobacterium smegmatis*. Conditions resulting in a significant reduction in titer compared to an untreated phage control at the same time point were determined by calculating the log change (log PFU_challenge_ − log PFU_control_) (see Supplemental File for Materials and Methods). All data from replicate experiments are presented in Table 1.

## Temperature

**Figure 1.**
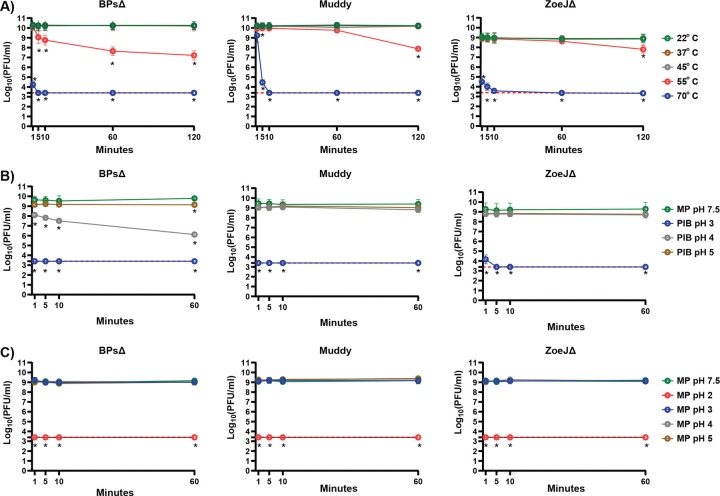
**A. Sensitivity of phages to various temperatures.** BPsΔ, Muddy, and ZoeJΔ were treated with a range of temperatures from to 22° C – 70° C. At specific time points, samples were removed and phages quantified. The mean of two independent experiments, each with three technical replicates, is plotted. Asterisks indicate significant reduction in log (PFU/ml) between a given treatment compared to the 22° C control at the same time point determined by one-way ANOVA (p<0.05) with Tukey’s post-test. **B. Effectiveness of Phage Inhibition Buffer (PIB) at various pH.** BPsΔ, Muddy, and ZoeJΔ were treated at a 1:10 ratio with PIB at pH 3, 4, and 5 and compared to MP Buffer pH 7.5. At specific time points, phages were quantified. The mean of two to three independent experiments, each with three technical replicates, is plotted. Asterisks indicate significant reduction in log (PFU/ml) between a given PIB pH time point compared to the MP buffer pH 7.5 control at the same time point determined by one-way ANOVA (p<0.05) with Tukey’s post-test. **C. Sensitivity of phages to various pH in MP buffer.** BPsΔ, Muddy, and ZoeJΔ were incubated in MP buffer at pH 2, 3, 4, and 5 and compared to MP Buffer pH 7.5. At specific time points, samples were removed and phage quantified. The mean of two to three independent experiments, each with three technical replicates, was plotted. Asterisks indicate a significant reduction in log (PFU/ml) between a given pH time point compared to the MP buffer pH 7.5 control at the same time point determined by one-way ANOVA (p < 0.05) with Tukey’s post-test. The red dashed line indicates the limit of detection. Error bars indicate standard deviation. Samples with zero PFU recovered were plotted at the level of detection.

Sensitivity to elevated temperature was monitored over a 120-minute (min) time course and compared to room temperature (22°C) (Fig 1A). At 37°C and 45°C there was no significant reduction in titer for any of the phages. However, with 55°C there was a time dependent reduction in all the phages, with BPsΔ exhibiting the greatest sensitivity. All three phages were highly sensitivity to 70°C.

## Ethanol

Sensitivity to 63% and 90% ethanol, a commonly used disinfectant, was tested (Table 1). All three phages were highly sensitive to 90% ethanol with undetectable levels at 1 min. 63% ethanol also had a rapid, although incomplete, effect with BPsΔ again exhibiting the greatest sensitivity.

## Phage inactivation buffer (PIB) and acidic pH

When measuring phage bactericidal activity by reductions in CFU it is advantageous to inactivate free phage before CFU plating. We previously reported use of a 10 minute treatment with a Phage Inactivating Buffer (PIB) (940 mM citric acid, 10 mM potassium chloride, 135 mM NaCl, pH 3.0) to inactivate BPsΔ, Muddy, and ZoeJΔ (7–9). Importantly, the host bacteria of these phages *Mycobacterium abscessus* and *M. smegmatis* are not inhibited by PIB pH 3, 10 min (8) (Supplemental Fig 1). To determine the time-dependence of inactivation, we performed a PIB time course and observed rapid reduction of the phages to undetectable levels: by 1 min for BPsΔ and Muddy and 5 min for ZoeJΔ (Fig 1B). The effect of pH could be important (10, 11). Therefore, we investigated the adjustment of PIB to either pH 4 or pH 5. Compared to PIB pH 3, PIB pH 4 and 5 had no effect on Muddy or ZoeJΔ, and PIB pH 4 and 5 was less effective at reducing BPsΔ (Fig. 1B). BPsΔ was again more sensitive than the other phages. We also studied the impact of acidic pH in MP buffer. All the phages were highly sensitive to MP buffer pH 2 (i.e., undetectable at 1 min) and resistant to MP pH 3, 4, and 5 (Fig. 1C). Thus, PIB pH 3 fully inactivates but MP pH 3 does not, which indicates that the pH of PIB is important but not the sole factor responsible for phage inhibition.

## Ferrous ammonium sulfate (FAS) +/− tannic acid (TA)

FAS +/− TA is another reported phage inactivating treatment (12–14). After 1 min incubation in 2.25 mM FAS with 0.002% TA the titer of all three phages was below the level of detection (Table 1). One min treatment with 2.25 mM FAS lacking TA also significantly reduced phage titers with Muddy being undetectable and BPsΔ and ZoeJΔ exhibiting a 4–5 log reduction. Treatment with 0.002% TA for 3 min had no effect (Table 1).

## Mucus

Several patients receiving phage therapy for pulmonary NTM disease have muco-obstructive lung diseases (2). Studies of the effect of mucus on other phages reveal inhibitory or beneficial effects (15–17). This led us to test the effect of mucus harvested from cultured human bronchial epithelial (HBE) cells (18, 19). Phages were incubated for 24 hours with 2%, 4%, or 6% mucus, which are concentrations that model healthy, moderate, and severe airway disease mucus (20, 21). Across the concentrations, all three phages exhibited either modest (≤0.7 log) or no significant reduction in titer. This finding of little to no effect of mucus supports the therapeutic use of these phages in people with muco-obstructive diseases.

## Reducing agents

Reducing agents that disrupt disulfide bonds degrade the mucin network of mucus. There is interest in developing reducing agents into mucolytics for muco-obstructive diseases (21, 22). At concentrations that reduce the viscosity of mucus, we tested reducing agents Tris(2-carboxyethyl)phosphine (TCEP) and Dithiothreitol (DTT) on phage activity (23). Because TCEP and DTT solutions are acidic, we tested each agent as an unbuffered solution and at pH 7. Without buffering, both 10 mM TCEP pH 1.9 and 1 mM DTT pH 5.3 after 24 hours significantly reduced the level of all three phages. However, both agents at pH 7 had no effect, apart from a modest reduction in ZoeJΔ PFU with 1mM DTT (Table1). Because mucus has a high buffering capacity, which limits pH effects *in vivo* (21), these results suggest that concurrent treatment with reducing mucolytics will not inhibit these phages.

## Detergents

**Figure 2.**
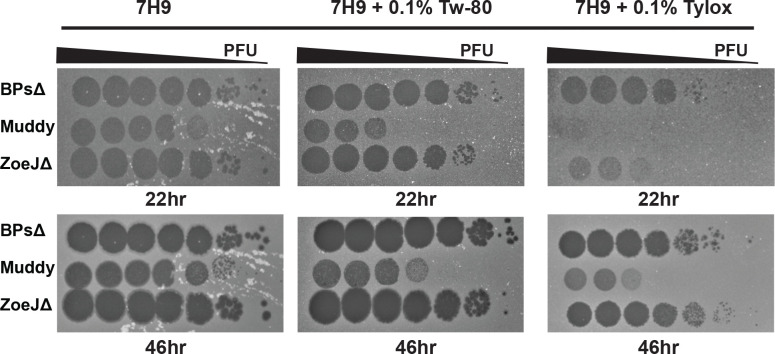
Sensitivity of phages to inclusion of detergent in the M. smegmatis culture used in agar overlays. BPsΔ, Muddy, and ZoeJΔ 10-fold serial dilutions spotted onto agar overlays prepared with M. smegmatis resuspended in Middlebrook 7H9 media supplemented with 0.5% glycerol, 0.2% glucose (no detergent) or the same Middlebrook 7H9 media with freshly added 0.1% Tween-80 (Tw-80) or 0.1% Tyloxapol (Tylox). Plates were incubated at 37° C for 22 and 46 hours. A representative image from two experiments is shown. All images were taken with the same exposure time of 0.09 seconds using a Bio-RAD GelDOC Go.

Sensitivity to detergents encountered in mycobacterial experiments (Tween-80, Tyloxapol, Triton X-100) was investigated. Tween-80 and Tyloxapol are included in mycobacterial liquid media to promote dispersed growth. Triton X-100 is used to lyse cultured eukaryotic cells infected with mycobacteria (8). All three phages were resistant to 60 min incubation with 0.1% Tween-80, 0.1% Tyloxapol, or 0.1%, 1%, or 10% Triton X-100 (Table 1). This resistance was surprising since Tween-80 is reported to prevent mycobacteriophage D29 infection (24). We wondered if the detergents might not inhibit the phage directly but, rather, prevent phage infection through effects on the mycobacterial host. To test this idea, immediately prior to use, 0.1% Tween-80, 0.1% Tyloxapol, or no detergent was added to the *M. smegmatis* culture used in the overlay for phage quantification (Fig 2). For BPsΔ and ZoeJΔ, Tween-80 or Tyloxapol in the culture did not affect PFU number although Tyloxapol delayed the time for ZoeJΔ plaques to appear. In contrast, inclusion of Tween-80 or Tyloxapol in the culture inhibited Muddy PFU. These results demonstrate that the activity of some, but not all, phages are impacted by detergent-specific effects on mycobacteria.

## DMSO

Many antimycobacterial drugs are dissolved in DMSO, such that 1% DMSO is likely to be included when phage-antibiotic combinations are tested on mycobacteria. DMSO may also be used as a protectant in long-term phage storage (25). Up to 72 hours, all three phages were resistant to 1% dimethyl sulfoxide (DMSO) (Table 1).

In conclusion, in studying three mycobacteriophages from phage therapy cases (2) we discovered several conditions that result in dramatic and rapid reductions in phage activity. We also observed differences between phages emphasizing the need for such studies. The results provide valuable information to support further evaluation of these phages as therapeutics for NTM disease.

## Supplementary Material

Supplement 1

Supplement 2

## Figures and Tables

**Table 1:** Mycobacteriophage Sensitivities (Log PFU/ml_Treatment_ - Log PFU/ml_Control_)

Condition	Time	BPsΔ	Muddy	ZoeJΔ
37°C	1, 5, 10, 60, 120 min	(NS, NS)	(NS, NS)	(NS, NS)
45°C	1, 5, 10, 60, 120 min	(NS, NS)	(NS, NS)	(NS, NS)
55°C	1 min	(NS, NS)	(NS, NS)	(NS, NS)
55°C	5 min	**(−1.09, −1.27)**	(NS, NS)	(NS, NS)
55°C	10 min	**(−1.47, −1.47)**	(NS, NS)	(NS, NS)
55°C	60 min	**(−2.64, −2.59)**	(NS, NS)	(NS, NS)
55°C	120 min	**(−2.97, −3.12)**	**(−1.58, −2.11)**	**(−1.04, −1.16)**
70°C	1 min	**(−5.40, −6.74)**	**(−1.90, −1.03)**	**(−4.13, −4.99)**
70°C	5 min	**(BLD, BLD)**	**(−5.84, −5.72)**	**(−4.90, −5.13)**
70°C	10 min	**(BLD, BLD)**	**(BLD, BLD)**	**(−4.78, BLD)**
70°C	60 min	**(BLD, BLD)**	**(BLD, BLD)**	**(BLD, BLD)**
70°C	120 min	**(BLD, BLD)**	**(BLD, BLD)**	**(BLD, BLD)**
90%EtOH	1, 5, 10, 60min	**(BLD, BLD)**	**(BLD, BLD)**	**(BLD, BLD)**
63% EtOH	1 min	**(−5.56, −5.13)**	**(−4.36, −2.16)**	**(−2.93, −2.42)**
63% EtOH	5 min	**(−6.47, −6.14)**	**(−4.72, −3.75)**	**(−3.80, −2.68)**
63% EtOH	10 min	**(BLD, −6.28)**	**(−4.36, −4.28)**	**(−3.14, −3.28)**
63% EtOH	60 min	**(BLD, BLD)**	**(−3.97, −4.79)**	**(−4.74, −5.19)**
1:10 PIB pH 3	1 min	**(BLD, BLD)**	**(BLD, BLD)**	**(−6.19, −4.55)**
1:10 PIB pH 3	5 min	**(BLD, BLD)**	**(BLD, BLD)**	**(BLD, BLD)**
1:10 PIB pH 3	10 min	**(BLD, BLD)**	**(BLD, BLD)**	**(BLD, BLD)**
1:10 PIB pH 3	60 min	**(BLD, BLD)**	**(BLD, BLD)**	**(BLD, BLD)**
1:10 PIB pH 4	1 min	**(−1.71, −1.86)**	(NS, NS)	(NS, NS)
1:10 PIB pH 4	5 min	**(−2.02, −2.08)**	(NS, NS)	(NS, NS)
1:10 PIB pH 4	10 min	**(−2.43, −2.30)**	(NS, NS)	(NS, NS)
1:10 PIB pH 4	60 min	**(−3.78, −3.67)**	(**−0.53**, NS)	(NS, NS)
1:10 PIB pH 5	1 min	(NS, NS, NS)	(NS, NS)	(NS, NS)
1:10 PIB pH 5	5 min	(NS, NS, NS)	(NS, NS)	(NS, NS)
1:10 PIB pH 5	10 min	(NS, NS, NS)	(NS, NS)	(NS, NS)
1:10 PIB pH 5	60 min	**(−0.62, −0.66, −0.67)**	(NS, NS)	(NS, NS)
MP pH 2	1, 5, 10, 60 min	**(BLD, BLD)**	**(BLD, BLD)**	**(BLD, BLD)**
MP pH 2	24 hrs	**(BLD, BLD)**	**(BLD, BLD)**	**(BLD, BLD)**
MP pH 3	1, 5, 10, 60 min	(NS, NS)	(NS, NS, NS)	(NS, NS)
MP pH 4	1, 5, 10, 60 min	(NS, NS)	(NS, NS)	(NS, NS)
MP pH 5	1, 5, 10, 60 min	(NS, NS)	(NS, NS)	(NS, NS)
2.25 mM FAS+0.002% TA	1 min	**(BLD, BLD)**	**(BLD, BLD)**	**(BLD, BLD)**
2.25 mM FAS	1 min	**(−4.26, −4.55)**	**(BLD, BLD)**	**(−5.18, −5.23)**
0.002% TA	3 min	(NS, NS)	(NS, NS)	(NS, NS)
2% HBE Mucus	24 hrs	(NS, **−0.40**, NS)	(NS, NS)	(NS, NS)
4% HBE Mucus	24 hrs	(**−0.15, −0.32**, NS)	(NS, NS)	(NS, NS)
6% HBE Mucus	24 hrs	(**−0.40, −0.48, −0.69**)	(NS, **−0.60**)	(NS, NS)
10 mM TCEP pH 1.9	24 hrs	**(BLD, BLD)**	**(BLD, BLD)**	**(BLD, BLD)**
10 mM TCEP pH 7	24 hrs	(NS, NS)	(NS, NS)	(NS, NS)
1 mM DTT pH 5.3	24 hrs	**(−5.2, −5.0, −5.2, −5.0)**	(**−0.32, −0.56**)	**(−3.67, −3.41)**
1 mM DTT pH 7.0	24 hrs	(NS, NS)	(NS, NS)	**(−0.45, −2.64)**
0.1% Tween 80	1 min	(NS, NS)	(NS, NS)	(NS, NS)
0.1% Tween 80	5 min	(NS, NS)	(NS, NS)	(NS, NS)
0.1% Tween 80	10 min	(NS, NS)	(NS, NS)	(NS, NS)
0.1% Tween 80	60 min	(NS, NS)	(NS, NS)	(NS, **−0.57**)
0.1% Tyloxapol	1, 5, 10, 60 min	(NS, NS)	(NS, NS)	(NS, NS)
0.1% Triton X-100	1 min	(NS, NS, NS, NS)	(NS, NS, NS, NS)	(NS, NS, NS, NS)
0.1% Triton X-100	5 min	(NS, NS, NS, NS)	(NS, NS, NS, NS)	(NS, NS, NS, NS)
0.1% Triton X-100	10 min	(NS, NS, NS, NS)	(NS, NS, NS, NS)	(NS, NS, NS, NS)
0.1% Triton X-100	60 min	(NS, **−1.46**, NS, NS)	(NS, NS, NS, NS)	(NS, NS, NS, NS)
1% Triton X-100	1 min	(NS, NS)	(NS, NS)	(**−0.52**, NS)
1% Triton X-100	5 min	(**+0.45**, NS)	(NS, NS)	(NS, NS)
1% Triton X-100	10 min	(NS, NS)	(NS, NS)	(NS, NS)
1% Triton X-100	60 min	(NS, NS)	(NS, NS)	(NS, **−0.67**)
10% Triton X-100	1, 5, 10, 60 min	(NS, NS)	(NS, NS)	(NS, NS)
1% DMSO	1 min	(NS, NS)	(NS, NS)	(NS, NS)
1% DMSO	5 min	(NS, NS)	(NS, NS)	(NS, NS)
1% DMSO	10 min	(NS, NS)	(NS, **−0.56**)	(NS, NS)
1% DMSO	60 min	(NS, NS)	(NS, NS)	(NS, NS)
1% DMSO	24 hrs	(NS, NS)	(NS, NS)	(NS, NS)
1% DMSO	48 hrs	(NS, NS)	(NS, NS)	(NS, NS)
1% DMSO	72 hrs	(NS, NS)	(NS, NS)	(NS, NS)

Bold text indicates significant difference compared to control based on (i) one-way ANOVA and Tukey’s post hoc (p < 0.05) or (ii) Unpaired t-test for conditions with a single time point (p < 0.05). NS = No significant difference. BLD = Below limit of detection [2,500 = PFU/ml; Log (PFU/ml) =3.5].

## Data Availability

Data from this study will be made available through Dryad upon acceptance.
